# Vulnerable older people’s views on proactive care planning: a qualitative interview study in primary care

**DOI:** 10.3399/BJGPO.2024.0167

**Published:** 2025-03-26

**Authors:** Lisa Kastbom, Anna Olaison, Annette Sverker, Anna Segernäs

**Affiliations:** 1 Department of Health, Medicine and Caring Sciences, Linköping University, Linköping, Sweden; 2 Primary Health Care Centre, Ekholmen, Linköping, Sweden; 3 Department of Culture and Society, Linköping University, Linköping, Sweden; 4 Department of Activity and Health, Linköping University, Linköping, Sweden; 5 Pain and Rehabilitation Center, Linköping University, Linköping, Sweden

**Keywords:** qualitative research, older adults, aged, primary health care

## Abstract

**Background:**

Patients in old age often have complex care needs owing to multimorbidity and polypharmacy. This qualitative study is part of a larger ongoing Swedish intervention trial Secure And Focused primary care for older pEople (SAFE), including shorter care agreements based on person-centred patient goals.

**Aim:**

To explore, in a primary care setting, the views of older and vulnerable patients on a more systematic, proactive approach to care planning, including establishing and documenting care agreements based on person-centred goals.

**Design & setting:**

Individual semi-structured interviews with patients (*n* = 25) aged >75 years from 12 intervention primary healthcare centres in two counties in Sweden.

**Method:**

Interviews were conducted between June and October 2023. They were digitally recorded and transcribed verbatim. Latent qualitative content analysis was used.

**Results:**

The following three categories, with 10 sub-categories, were found: I would like to live in the present, so why plan ahead? Let me decide versus they know best; and Care agreements usually went unnoticed. The latent theme — The ambivalence of care planning in the fourth age — was created to give a deeper meaning to the content of the categories.

**Conclusion:**

This study emphasises that older, vulnerable persons have varying attitudes towards participation in proactive care planning. This ambivalence may originate from the individual’s desire to have their autonomy respected and express future care preferences on the one hand, and to avoid or postpone end-of-life conversations and care planning on the other hand. Patients also expressed a desire to be more actively involved in care planning. Although care agreements have the potential to increase patient involvement in proactive care planning, they often went unnoticed. The conversation itself was essential.

## How this fits in

Person-centred care is applicable to all patient groups and has been shown to be particularly useful for individuals where care needs are complex, a frequently identified situation for older persons in primary care. This study emphasises that older, vulnerable persons have varying attitudes and preferences regarding how, and to what extent, they want to be involved in proactive care planning. Ambivalence towards care planning in the fourth age originated from the desire to respect autonomy and a desire to express preferences regarding future care on the one hand, and to avoid or postpone end-of-life conversations on the other hand. Documented care agreements often went unnoticed, but the conversation with committed healthcare staff created reassurance and trust in itself, although some older patients expressed a desire to be more actively involved in their care and appreciated proactive care planning.

## Introduction

The proportion of older people in the population is increasing, and the greatest need for care is seen among a small section of the population, often with a significant number of multimorbidities.^1,2^ A certain number of older people are vulnerable and have particularly high care needs, and some meet the criteria for frailty.^3,4^ International recommendations have underlined the importance of early identification and holistic assessment of people with frailty and an increased risk of inpatient care.^3,5^ For this patient group, the holistic form of care called 'Comprehensive Geriatric Assessment' (CGA) is important and regarded as the gold standard in hospital geriatric care.^6–8^

Person-centred care (PCC) is appropriate for all patient groups and has been shown to be particularly useful when care needs are complex, a situation frequently encountered in older patients.^9^ Previous research has shown that the individual’s needs, opportunities, and goals should be identified and highlighted,^9–11^ and it is valuable for healthcare staff to have personal knowledge of the factors that are important in PCC.^12^

The ongoing intervention trial Secure And Focused primary care for older pEople (SAFE) is a follow-up and extension of the previous study *Proactive healthcare for frail elderly persons*.^13,14^ SAFE is intended to evaluate a customised, person-centred primary care multiprofessional CGA team model that can be widely implemented in general practice in close collaboration with surrounding healthcare providers. The intervention involves a validation prediction model that estimates the risk of hospitalisation for patients living in ordinary homes and aged from 75 years, combined with an evaluation by a specialised elder care team (in Swedish: *äldrevårdsmottagning*) at each primary healthcare centre (PHCC).^13,15^ Individuals living in nursing homes were excluded from participation. The elder care teams consist of a nurse and a physician, most often a general practitioner (GP), who apply the CGA team model to evaluate each patient by using the Primary Care Assessment Tool for the Elderly (PASTEL).^16^ Coordination between primary care, home nursing, and rehabilitation (at home and in practices) is reinforced during the 2-year follow-up period. The objective is to obtain additional data on the large-scale implementation of the model and to evaluate its cost-effectiveness in a larger primary care population in several Swedish regions, as well as to study the experience of increased participation of patients and their relatives in care and proactive care planning.

Previous research has shown that approximately 50% of older adults have a documented care plan and few talk to their primary care provider about end of life (EOL).^17^ There is a need to evaluate strategies used in primary care settings to initiate advance care planning (ACP) conversations. Primary care physicians working in practices characterised by an advanced primary care model, including a patient–team partnership, comprehensive and coordinated care, continuity of care, and team-based care, are more likely to initiate and document ACPs.^18,19^ However, as far as we know, knowledge is scarce regarding what actually benefits the patients themselves, and what kind of proactive care planning and involvement in care older persons perceive to be of use in a primary care setting.

ACP is a proactive communication process,^20^ supporting adults at any age or health status to understand and share their personal values, life goals, thoughts on life and death, and preferences for future medical care.^20,21^ There is consensus that older frail adults express an interest in ACP conversations, but only a small proportion of older patients actually have a plan.^22^ To what extent systematic ACP might have beneficial effects in terms of future care planning for a wider primary care population with older, vulnerable patients remains to be investigated.

This qualitative interview study is part of the intervention study SAFE in Swedish primary care, which aims to apply the positive factors described for practices that work more routinely with ACPs. As part of the primary care CGA working model in the study protocol, ACP conversations, documentation, and shorter care agreements, that is, documented agreements based on person-centred patient goals identified together with the elder team nurse, are used more routinely with both patients and family members. In Sweden, there is a national decision to introduce work with proactive care agreements in general, not just in primary care. The elder team nurses, who are part of the ordinary staff at the PHCCs, are the regular clinical care contact for the older patients and are not research study nurses.

The aim of this qualitative interview study was to explore, in a Swedish primary care setting, the views of older, vulnerable patients on a more systematic and proactive approach to care planning, including establishing and documenting care agreements based on person-centred goals.

## Method

### Participants and interviews

Inclusion criteria were: participants should be patients at one of the 26 intervention PHCCs in the two county councils (Östergötland or Jönköping) in the SAFE intervention trial; be Swedish speaking; and consent to the recording of interviews. Participants were recruited using purposive sampling.^23^ Exclusion criteria were: suffering from severe psychiatric illness; and having cognitive impairment and/or severe hearing loss that would prevent participation in an interview. All participants were living in ordinary homes, with or without municipal care. The responsible nurses at the intervention PHCCs were asked to identify potential participants who had been seen for PASTEL assessment and followed-up by the nurse after the start of the intervention. The nurses assessed whether the selected patients were able to participate in an interview according to the inclusion and exclusion criteria. Only those who were assessed by the nurses as able to participate were asked to take part in the study. Patients with a documented diagnosis of dementia in their medical records were excluded. In cases where the nurse suspected that there was a cognitive impairment during the clinical assessment, while history taking, and/or due to symptoms (PASTEL also includes a question about memory difficulties), the nurse refrained from asking about potential study participation in this qualitative study. Of the 28 individuals who were asked by the research team to participate in the study, three declined participation. The main reason for not participating was poor health. The mean age of the participants was 84 years (range 76–92). Fourteen (56%) were women, and 11 (44%) were men. Educational level and socioeconomic status of the participants varied.

An interview guide was developed by the research group (see Supplementary Appendix S1). The interviews were conducted between June and October 2023 by three researchers, consisting of a GP (LK: female, PhD, MD) and two social workers (ASv and AO: both females, PhDs and associate professors), all with experience in qualitative research from different scientific disciplines such as health, ageing, and social work research. Twenty-five participants were interviewed individually (see Table 1). Common characteristics of the participants were old age, multimorbidity, and varying degrees of frailty. The interviews (length varied between 29 and 108 minutes) were conducted by telephone (*n* = 15) or face-to-face (*n* = 10) in the participant’s home, depending on the participant’s preference. Participants were included until saturation occurred, that is, conducting additional interviews was not considered to add new views and perceptions.^23^ Interviews were digitally recorded and transcribed verbatim. Transcripts were not returned to participants for comments and/or corrections.

**Table 1.** Characteristics of the 25 participants

**Characteristic**	***n*** **(%)^a^**
**Age, years, mean (range)**	84 (76–92)
**Gender**	
Man	11 (44)
Woman	14 (56)
**Living situation**	
Cohabitant	15 (60)
Living alone	10 (40)
**Relationship status**	
Married	22 (88)
Widow/widower	7 (32)
Not married	3 (12)
**Primary healthcare centre county**	
Östergötland	8 (67)
Jönköping	4 (33)
**Primary healthcare centre setting**	
Rural	5 (42)
Urban	7 (58)

^a^Unless otherwise stated.

### Analysis

**Figure 1.**
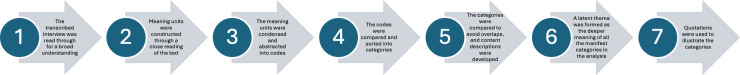
The seven steps of the latent qualitative content analysis used in the analysis

The interviews were analysed using latent qualitative content analysis. This method allows for an open-ended analysis with no preconceived categories, allowing the categories to emerge from the data.^24^ Figure 1 shows the steps used in the analysis. The preliminary categories were coded mainly by the first and second authors (LK and AO). Validity was built into the analysis by testing the preliminary categories until saturation was reached by the entire research group. As part of the reflexivity process, the categories were confirmed by complementing and contesting each other’s readings and pre-understandings.^25^

## Results

**Figure 2.**
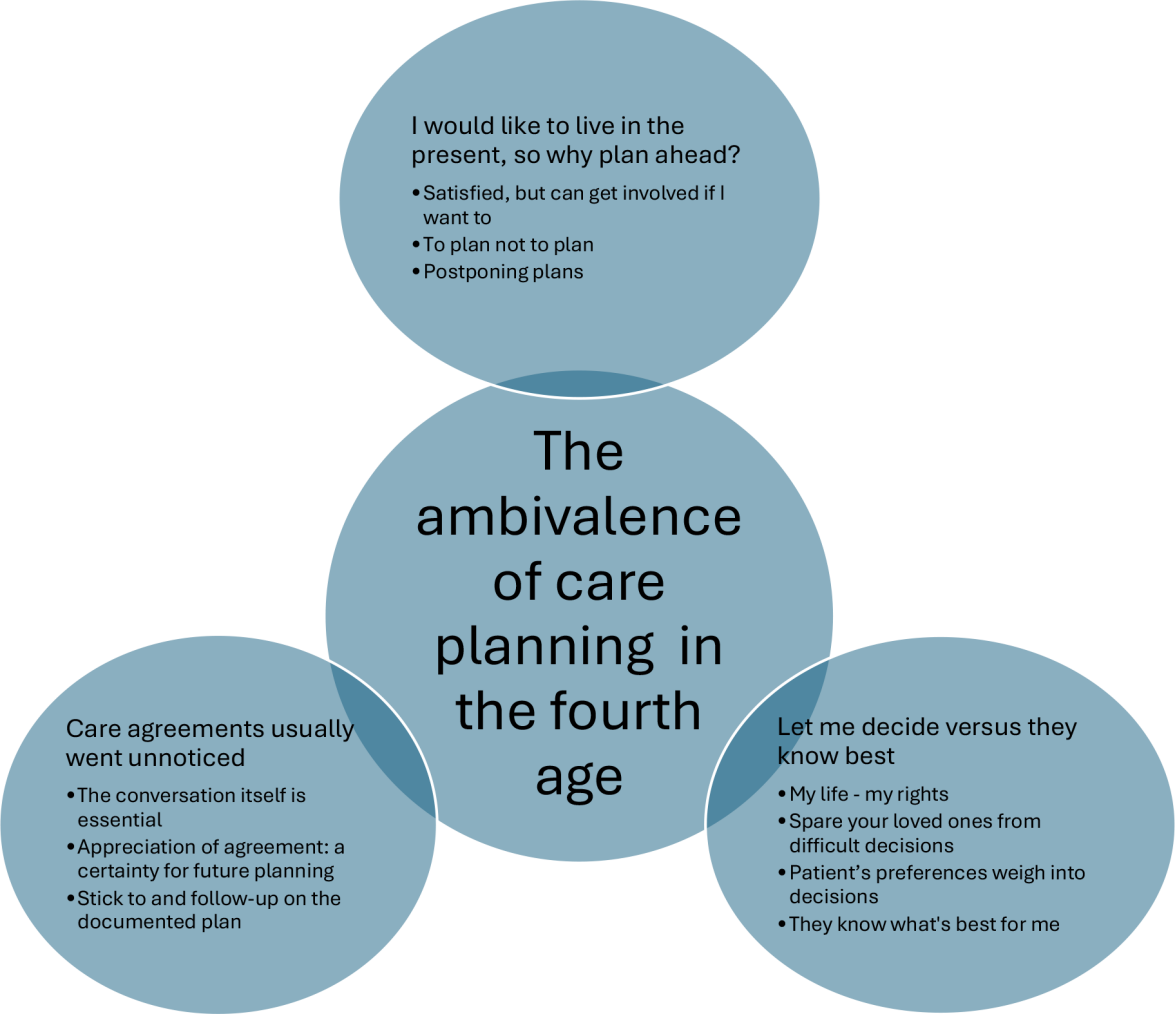
Overview of the three categories with 10 sub-categories and an overarching theme describing the views of older, vulnerable patients on a more systematic, proactive approach to care planning in a Swedish primary care setting

When analysing the data through latent qualitative content analysis,^24^ the following three categories describing older patients’ views on proactive care planning emerged: I would like to live in the present, so why plan ahead? Let me decide versus they know best; and Care agreements usually went unnoticed. These categories included 10 sub-categories. The overarching, latent theme — The ambivalence of care planning in the fourth age — was created to give a deeper meaning to the content of the categories (see Figure 2). Examples of the steps of the analysis are shown in Table 2.

**Table 2.** Examples of the steps of the analysis using latent qualitative content analysis

Meaning unit	Code	Sub-category	Category
*'I’m not worried, no, because everyone has to change their lives more or less when they get old and can't manage on their own. So I'm prepared for that, that the day will come when I have to do that. And that’s a concern for another day. I'll think about it then, but I won't worry about it now.'*	Not worried about the future One day, you can’t manage on your own A concern for another day	Postponing plans	I would like to live in the present, so why plan ahead?
*'It’s documented … In the medical records. So, it’s no harder than having someone else look it over and take over if someone quits or something like that. Then what we have agreed upon is documented. So, it can be an advantage*.'	Appreciation of documented agreement Agreement between patient and healthcare staff An advantage when healthcare staff can read the documented agreement	Appreciation of agreement: a certainty for future planning	Care agreements usually went unnoticed
*'Yes, but if I'm* [cognitively] *coherent enough to express what I want, then you should respect that, I think. I absolutely think so. The day I can't object any longer, then my relatives have to take over and speak on my behalf*.'	Respect one’s will when cognitively clear Autonomy Relatives’ right to speak on patient’s behalf when not cognitively clear	My life, my rights	Let me decide versus they know best

### I would like to live in the present, so why plan ahead?

The participants’ attitudes towards active involvement in care planning varied. Some expressed that they want to live life in the present, but if something happened, they wanted to be involved in the planning on their own terms. Others had a strong desire to avoid or postpone, or did not want to plan or participate in care arrangements in a variety of ways.

#### Satisfied, but can get involved if I want to

A common theme among the participants was that some expressed a desire to live in the present and did not want to think about planning their future care. However, in these cases, the participants felt reassured that they could be involved if they wished, and they knew what actions they needed to take to be active in planning their own care. Patients who highlighted these views emphasised the value of proactive care planning. They appreciated the fact that there was a plan for their care that included future care needs:

*'Yes, they need to have that* [a care plan]*. Then they basically know what the patient wants. And if you're unconscious at a later stage, then they know what you want and ... Healthcare needs to benefit from proactive care planning.'* (Participant 5, man, aged 87 years)

#### To plan not to plan

Participants expressed their awareness of the finiteness of life and the uncertainty of the future, especially older participants who suffered from chronic diseases where a deterioration in health could be expected. Notwithstanding the awareness of the finiteness of life, there was no intention to actively participate in planning for care and support. This stemmed from the view that life should take its course without too much planning. Participants occasionally expressed a desire to know how long they had left to live, perhaps in order to make the most of the time they had left:

*'Yes. I mean, when you have the disease that I have got that is actually hereditary, which I know, my mother had it too, then you always have the shadow of death over you. And you want to somehow ... well, what can I say, be aware of how much time you have left. But you almost never get that. No, you can't. Because no one can say what will happen in an uncertain future*.*'* (Participant 18, man, aged 89 years)

#### Postponing plans

Uncertainty about the future did not always cause worry, and participants sometimes expressed that they lived for the moment, postponing thoughts of impairment and active planning for future care needs. Instead, they felt it was important to live for the moment, although there was an awareness that deterioration from old age and morbidity could come at any time. For those who were in adequate health, remaining active helped participants focus on the present and put thoughts of the future aside. Some of the participants felt that these issues should be dealt with at a later date when the need arose:

*'I’m not worried, no, because everyone has to change their lives more or less when they get old and can't manage on their own. So, I'm prepared for that, that the day will come when I have to do that. And that’s a concern for another day. I'll think about it then, but I won't worry about it now.'* (Participant 27, woman, aged 92 years)

### Let me decide versus they know best

Some participants had a strong desire for control and wanted to be actively involved in care planning, either personally or with the help of their relatives, while others had full trust in health professionals, leaving them to plan and make appropriate decisions about care and services.

#### My life, my rights

Participants expressed a desire to be involved in care by receiving clear information and feedback from healthcare staff. It could be a matter of receiving test results by post or receiving feedback from the physician over the phone. For one participant, the fact that a physician they had never met was well-informed about their medical history by reviewing their medical records created a sense of security and trust, and this level of communication contributed to a feeling of involvement.

*'I take a lot of* [blood] *tests there and then I want a little more specific, preferably a list that I can compare each time.'* (Participant 8, woman, aged 84 years)

Some participants expressed a wish to be involved in the planning and content of their care and for their autonomy to be respected, even in the last days of life. If they were no longer able to express their own wishes, they wanted their relatives to speak on their behalf:

*'Yes, but if I'm* [cognitively] *coherent enough to express what I want, then you should respect that, I think. I absolutely think so. The day I can't object any longer, then my relatives have to take over and speak on my behalf.'* (Participant 21, woman, aged 76 years)

#### Spare your loved ones from difficult decisions

Some of the participants had experience making decisions on care planning for a relative. Because of their own negative experiences and the distress it caused, they wanted to prevent their loved ones from ending up in the same situation. They therefore hoped to have the opportunity to express their own preferences if their health deteriorated:

*'I hope that I am able to discuss things then, because ... So that no one else has to decide for me. You never know, but I hope that ... I will be able to participate in the planning of care when that day comes.'* (Participant 1, woman, aged 78 years)

#### Patients' preferences weigh into decisions

For some, patient involvement in care and active care planning meant making decisions, based on discussions with the doctor, in which the patient’s own wishes were heard and taken into account. In these cases, participants described a scenario where patients and doctors help each other to make different decisions about the content of care:

*'Well, it’s that we should be able to talk to each other, and I can talk about what I feel and that they really understand me, that they can share it with me, so that they understand that I really need this and that. For me, I guess that is participation.'* (Participant 4, woman, aged 88 years)

#### They know what’s best for me

Other participants relied on the healthcare system, believing that healthcare professionals were best suited to make medical decisions and plan care in the most appropriate way. They expressed gratitude for not having to be involved in decision making and felt a sense of trust:

*'Yes, I don't feel like I need to be ... nothing but being there, and then they take care of me. I'm not involved very much in that case.'* (Participant 9, man, aged 87 years)

### Care agreements usually went unnoticed

Care agreements were central to the person-centred primary care CGA working model when planning care for each individual patient. However, the process of setting up care agreements went relatively unnoticed by the participants. In particular, the process of setting up an agreement and documenting this occurred without the patient’s knowledge. However, participants reported that discussing the agreement with the nurse and going through their care needs and planning was a valuable experience. Most participants did not realise that a documented agreement had been made.

#### The conversation itself is essential

Although the care agreement based on person-centred goals is central to the care process for the nurse in the elder care team, the agreement often went unnoticed. What the study participants highlighted as important was the actual discussion with the nurse. They appreciated meeting a nurse who had set time aside, who listened, and who saw the whole person. This gave a sense of security and built trust. According to the participants, old age is often associated with the feeling of being a burden to those around you. A nurse who created a sense of security and trust made them feel important:

*'Yes, I feel that I'm important in some way, that I’m the one that they're taking care of and it’s about me when I'm there. That, I feel.'* (Participant 20, woman, aged 77 years)

Some argued that creating and documenting care agreements was just an administrative burden for staff. This was seen as unnecessary and something that was done at the expense of other issues that were more important for health care. They felt that owing to productive conversations, healthcare staff already knew the patient’s wishes and preferences.

[On creating and documenting care agreements] *'Well, I don't know if I would gain anything from that, but I think it might be good to know it. But besides that, it’s nothing that I need. No, it’s probably unnecessary work, I think ... Yes, there are so many other things you could do in health care. You shouldn’t need to ... You know exactly how I feel already, so there’s no reason to attend to that medical records stuff. No, no, I don't think so.'* (Participant 4, woman, aged 88 years)

#### Appreciation of agreement: a certainty for future planning

Others felt that the agreement could be of value, at least to healthcare professionals, and that it could provide indirect reassurance to patients to know that the care plan and agreement were documented in the patient’s medical record, for example, in cases where healthcare professionals were involved other than those to whom they had expressed their wishes and attitudes:

*'It’s documented … In the medical records. So, it’s no harder than having someone else look it over and take over if someone quits or something like that. Then what we have agreed upon is documented. So, it can be an advantage.'* (Participant 21, woman, aged 76 years)

#### Stick to and follow-up on the documented plan

Sometimes, participants had a clear opinion about care restrictions in the event of sudden deterioration, such as withholding cardiopulmonary resuscitation. Although this was documented in one patient’s medical records, they carried a written note in their wallet as extra assurance that their wishes would be respected:

*'… that they're not trying to restart my heart. And in my wallet … I put a note. My friends know about it, that there is a note there. And that it says, "Please, when my heart has stopped, let it stay stopped."'* (Participant 13, woman, aged 82 years)

There were limitations to the participants’ experiences of care plans. One had experience making a care plan for a loved one, where the conversation that preceded the care plan was perceived as positive and the plan itself created a sense of security. However, according to the participant, this feeling of security turned into disappointment when the content of the care plan was not followed:

*'Overall, you shouldn't plan at all if you can't stick to the plans you've made, because I've been through that too ... Yes, I would, of course, like to be with it until the end and understand these nice instructions that I received here at this meeting and the nice conversation. But in hindsight, I think "why do they say this when it doesn't turn out that way?" It’s just fairy tales, everything … '* (Participant 17, woman, aged 87 years)

The participant here emphasises the importance of healthcare professionals being able to implement and complement care plans, otherwise they see no point in doing so.

### The ambivalence of care planning in the fourth age

When analysing the interviews, the latent theme — The ambivalence of care planning in the fourth age — was formed to provide a deeper understanding of the content of the three categories, with sub-categories describing older and vulnerable patients’ views on a more systematic and proactive approach to care planning in a Swedish primary care setting (see Figure 2).

The older persons participating in this study expressed varying attitudes towards being involved in planning the content and direction of their future care. Some wanted to be involved to a greater extent and asked for their preferences and values to be respected, while others preferred to let others, such as healthcare professionals and/or family members, decide on these questions.

When planning care in later life, participants felt it was important to have conversations with the elder team nurse, as this was the healthcare provider who saw the whole person and had time to listen to the older person’s thoughts. Such conversations created reassurance and trust, which are cornerstones of conversations about EOL issues in proactive care planning. The conversation itself seemed more important to older people than the documentation of care agreements and ACPs in the patient’s medical record. This was often seen as a purely administrative task for healthcare staff, although some participants saw the value of documenting the plan, provided that the plan was followed.

## Discussion

### Summary

This study emphasises that older, vulnerable persons have varying attitudes towards being involved in the proactive planning of their future care. Ambivalence towards care planning in the fourth age emerged, on the one hand, from the individuals’ desire to have their autonomy respected and their will to be able to express their own preferences about their future care; on the other hand, the desire to avoid or postpone EOL conversations and care planning, so that they could continue to live in the moment. Other aspects contributing to uncertainty around proactive care planning originated from a focus on the care dialogue itself, where engaged healthcare staff created a sense of reassurance and trust, while the documentation of plans was often considered a purely administrative task. While some individuals in this patient group seemed to have a strong desire for control and preferred to be actively involved in care planning, others had complete trust in healthcare staff, leaving the planning and decisions about the content and direction of care to the professionals.

### Strengths and limitations

Dependability was reached as the audit trail could easily be followed through the entire process.^23^ Data were collected from PHCCs from two counties, which could be seen as data triangulation.^23^ Four researchers were involved in the analysis of the data, strengthening the results through investigator triangulation and ensuring credibility.^23^ Sampling was purposive because the responsible nurses at the intervention PHCCs were asked to identify potential participants. This could be seen as a limitation; however, the nurses were instructed to ask potential participants who had been seen for a PASTEL assessment and followed-up by the nurse after the start of the intervention, preferably in a specific order. Patients with severe hearing loss were excluded from the study, which could be seen as a limitation, as this group represent a significant proportion of older adults, and as having severe hearing loss makes interactions with healthcare staff challenging this would have been an important perspective. As this study is a qualitative interview study, it is necessary to have sufficient hearing and language to be able to conduct an interview. However, in further studies this important group of patients could be included by collecting data from their written thoughts. In the present study, the interviews were conducted by three researchers: one GP and two social workers, all with rich experience in qualitative research from different scientific disciplines, such as health, ageing, and social work research. A researcher without personal knowledge of the participants conducted the interviews. One of the social workers who conducted interviews had long and genuine experience in clinical work in a healthcare setting where consultations with older, vulnerable patients are part of the daily work. In those cases where the researcher who was a GP with experience of caring for older patients in primary care conducted the interviews, this information was disclosed to the participant, who was also told that the primary role of the interviewer in the interview situation was as a researcher, who was aware of preconceptions and aimed to remain neutral throughout the study process. Although this may have influenced the answers of the participants to some extent and is therefore seen as a limitation, the strengths of involving researchers with different scientific backgrounds and professions are considered greater, as the interview approach is likely to have resulted in a broad range of issues being highlighted, thus adding greater depth to the data. All four researchers in the research group were involved in the analysis and the results were discussed by the whole research group until agreement was reached, as part of the validation of the findings, which contributed to trustworthiness.^23^

### Comparison with existing literature

Primary care physicians face barriers in engaging patients in conversations about prognosis, values, and goals, and there is interest in finding structured tools to facilitate these conversations in primary care.^26^ Different approaches have been evaluated to identify patients who would benefit from proactive care planning, but GPs continue to face challenges when attempting to start conversations about future care.^1,27^ In the Swedish SAFE trial, we are implementing shorter care agreements based on person-centred goals identified together with the elder team nurse. This shorter care agreement, sometimes used together with an ACP, is one part of the SAFE primary care adapted CGA model, which is an approach that can potentially be used to routinely engage patients and family members in conversations and proactive care planning. However, the present study found a discernible ambivalence regarding patients’ perceptions towards proactive care planning and to what extent documentation is necessary, highlighting the need to approach the conversation itself in a person-centred manner.

### Implications for practice

Care agreements are new tools in the SAFE working model, usually occurring at an earlier stage than ACPs, and are therefore different from the usual routine of ACPs involving EOL issues. These agreements can potentially be used to increase patient involvement in their care and in the proactive planning of their care. However, more research is needed on the interaction between patients, relatives, and healthcare staff around care agreements in clinical practice. Patients show a certain ambivalence in their attitudes towards proactive care planning and the extent to which they feel documentation is necessary. Furthermore, patients report that it is the care planning conversation itself that is the most important aspect and that proactive care planning needs to be approached in a person-centred way. Systematic work with care agreements and ACPs that relies on a CGA model, which is adapted to primary care, could be a way to help primary care staff initiate a conversation with patients and relatives about future care, including EOL questions.

We believe that it is possible to find transferability of our findings to similar groups of older, vulnerable patients in primary care. In Sweden, there is a national decision to introduce work with proactive care agreements overall, not just in primary care. Prioritised patient groups for establishing care agreements are older, vulnerable persons, and patients with multimorbidity and complex diseases. Different perspectives on the ambivalence of care planning in the fourth age need to be visualised, not only in Sweden, but also internationally. Proactive care planning is sometimes challenging in the CGA team model, which is considered the gold standard in holistic geriatric care. Patients’ desire to live in the present is sometimes in contrast to healthcare professionals’ ambitions to plan ahead.

## Trial registration number

The SAFE study is registered at https://clinicaltrials.gov (identifier: NCT05706272).
